# Medical Society of the County of Erie

**Published:** 1892-04

**Authors:** Eli H. Long

**Affiliations:** Secretary


### MEDICAL SOCIETY OF THE COUNTY OF ERIE.

Reported by ELI H. LONG, M. D., Secretary.

*Special Meeting held in the Y. M. C. A. Building, Feb. 20, 1892.*

The President, William Warren Potter, M. D., in the Chair.

The meeting was called to order at 8.30 p. m., and the President stated the object of the meeting, which had been called at the request of five members, to take action with reference to the Bill pending in the State Legislature providing an amendment to the Medical Examiners' Law of 1890, exempting certain students from its operations. He gave a résumé of the origin of the movement for State license and the struggles to obtain the law, and stated that it was now in a fair way to be rendered useless, for a time at least, if the amendments prevailed that were now proposed and advocated by the students of the classes of '93 in the several medical colleges, with the exception of that of Niagara University, which had pronounced against it in unmistakable terms.

Dr. CHARLES CARY moved that a committee of three be appointed by the chair to formulate a suitable expression of the opinion of the society on the subject. Drs. Charles Cary, Thomas Lothrop, and Edward Storck were appointed a committee in accordance with these instructions of the society, and after deliberation reported the following:

WHEREAS, The Medical Society of the County of Erie is, and has been from the earliest agitation of the subject, in favor of a separate State Examining and Licensing Board; and,

WHEREAS, This society took the initial action which led to the passage of the present law (Chapter 507, Laws of 1890,) on this subject; and,

WHEREAS, This society has heretofore, namely, on the 20th of March, 1891, expressed its opposition to any amendments of the law until it should be thoroughly tested; and,

WHEREAS, This society has heard that a bill for the exemption of students who matriculated in medical colleges prior to December 1, 1891, has already passed the Senate; and,

WHEREAS, This society has also learned that the same or a similar bill is now pending in the Assembly, known as Assembly bill No. 513; and,

WHEREAS, This society deems the passage of such a measure as directly prejudicial to the public interests of the State, in that it delays an attempt to improve the public health service, which was sought to be benefitted by giving to the people better educated and more amply equipped physicians; and,

WHEREAS, The faculties of the University of Buffalo and the Niagara University, the only medical teaching faculties in Western New York, are members of this society, and as such members in their individual capacity, as well as in their official relations to their respective medical colleges, are absolutely and unqualifiedly opposed to any amendments at present to the law in question; therefore,

*Resolved,* That we, the members of the Medical Society of the County of Erie, in special session assembled, do hereby request the Senator and Members of the Assembly from Erie county to use all honorable means to defeat any legislation which may have been presented, or which may hereafter be offered in either house of the Legislature, looking toward any change or modification in Chapter 507 of the Laws of 1890 as it now stands; and particularly do we request each and every one of our representatives in the State Legislature of 1892 to use their utmost endeavors to prevent the passage of the Bill referred to, now understood to be resting with the Committee on Public Health in the Assembly, and known as Assembly Bill No. 513.

CHARLES CARY,
THOMAS LOTHROP,
E. STORCK,
*Committee.*

After remarks by Drs. Cary, Lothrop, Hopkins, Storck, Clark, Mynter, and others, the preamble and resolution were adopted without dissent.

Dr. Henry R. Hopkins then offered the following memorial, which was approved by the society and ordered to be transmitted to the Assembly, signed by the President and the Secretary:

*To the Assembly:*

The Medical Society of the County of Erie, in special session convened, on the 20th day of February, 1892, respectfully petitions your honorable body to reject Assembly Bill 513, which provides certain amendments to Chapter 507 of the Laws of 1890, for the following reasons:

*First.*—Because the law referred to (Chapter 507, Laws of 1890,) is a beneficient measure, framed and enacted in the interest of the public, to promote public health, and to prevent unqualified men from exercising the functions of physicians.

*Second.*—Because it is a wise law, one in accordance with the spirit of the age, is in keeping with similar statutes prevailing in other States on the subject of separate licenses for physicians, and has been used as a model in many of the States. We are informed that some twenty-four States now provide that physicians shall not practise within their borders without a State license.

*Third.*—Because the law was passed after considerate and prolonged discussion and argument, not only among medical men, but by the public in general and through the newspaper press in particular, and it did not take effect until some fifteen months after it had received the sanction of the executive. It was signed June 5, 1890, to go into operation September 1, 1891.

*Fourth.*—Because already such students as have matriculated in the medical colleges in this State prior to June 5, 1890, have been exempted from the provisions of the law by a special act which amended it to that effect, passed last year.

*Fifth.*—Because we feel that it is prejudicial to the public welfare, in opposition to the spirit of the law, and against right, justice, and good government, to grant any further exemption, or to amend the existing statute in any particular, until it has been tested by at least a year's trial in its application to the graduates of colleges in the State of New York.

*Sixth.*—At present only those physicians who have received diplomas granted from without the borders of the State of New York are amenable to the provisions of the law, and we believe it unjust to compel the citizens of other States to undergo State examinations that are not also required of the citizens of the State of New York. Discrimination of this sort against the citizens of other States has always been considered contrary to the spirit of our fundamental law, and certainly was not the intent of the framers of the existing law on the subject of State examinations for State licenses.

*Seventh.* – For these and other reasons which might be named almost *ad infinitum*, but which we forbear to enumerate lest we needlessly take up too much of your valuable time, we pray you, in all earnestness, to grant our petition.

Dated at Buffalo, February 20, 1892.

For the Medical Society of the County of Erie.

(Signed), WILLIAM WARREN POTTER, *President.*

Attest: ELI H. LONG, *Secretary.*

On motion of Dr. Edward Clark, a committee of three, one of which shall be the President, and the other two to be appointed by the chair, was directed to proceed to Albany, and present the views of the Medical Society of the County of Erie to the Committee on Public Health, in opposition to Assembly Bill 513, known as the Students' Exemption Bill. The chair appointed as members of that committee, Dr. Edward Clark and Dr. Thomas Lothrop, but the latter having declined to serve by reason of professional engagements, Dr. Ernest Wende was substituted in his place.

Dr. HOPKINS moved that the Secretary address a circular to the several county medical societies throughout the State, inclosing the official proceedings of the society on this subject, and asking their coöperation to prevent the passage of the bill in question. After some remarks suggesting methods of procedure by several members, the motion was adopted without division.

The society then adjourned.

### SECOND SPECIAL MEETING.

Reported by DeLANCEY ROCHESTER, M. D., Secretary *pro tem.*

A special meeting of the Medical Society of the County of Erie was held at its hall, in the Y. M. C. A. Building, Monday, February 29, 1892, to take action with reference to the death of Dr. John D. Hill.

The President, William Warren Potter, M. D., called the meeting to order at 8.45 P. M., in a few remarks with reference to the life and character of the deceased. In the absence of the Secretary, Dr. DeLancey Rochester was appointed Secretary *pro tem.*

On motion of Dr. Thomas Lothrop, seconded by Dr. H. R. Hopkins, a committee of three was appointed by the chair to prepare a memorial expressive of the sentiments of the society with reference to the deceased. The chair appointed as members of this committee, Drs. Thomas Lothrop, F. W. Bartlett, and H. R. Hopkins. After deliberation the committee reported the following :

### MEMORIAL.

### JOHN DAVIDSON HILL, M. D.,

*Ex-President of the Medical Society of the County of Erie, Died Feb. 27, 1892, Aged 70 years.*

This society is called together in special meeting to record its deep sense of sorrow in view of the death of another of its oldest and most honored members. The subject of this memorial has been identified with the professional and eleemosynary interests of this city from his early manhood, and has at all times commanded the respect and confidence of his fellow-citizens for his eminent ability, his superior administrative qualities, and the energy and independence of his character.

The distinguishing feature of his life has been portrayed in his strong personality ; he possessed a wide and matured experience, rare judgment in practical medicine, and skill in his profession, which commanded a large and lucrative practice among the most substantial and intelligent members of the community.

He was thoroughly self-reliant, and his judgment at the bedside, his fertility of resource, his untiring devotion to his patients, as well as his self-sacrificing labors in their behalf, were sources of strength which have been recognized and appreciated among his friends and patrons.

He was universally respected as an upright citizen, a sagacious financier, a devotee to the highest moral and religious interests of the community in which he lived, and who gave according to his means and opportunity to the advancement of the varied interests of the city of his adoption, for which he entertained a commendable pride.

Failing to recognize the fact that increasing years bring a decrease of physical vigor which his professional experience and perception detected in others, he devoted to his practice, even after passing the meridian of life, the same tireless energy and self-sacrificing labor which were required of him in the full vigor of his manhood. Exhausted strength failed to find adequate recuperative powers, and he awakened to the reality, too late to correct the mistake which became apparent to him, that his physical forces were undermined, and fatal disease had gained the mastery over the robust and vigorous organization which had stood him well in hand in the activities of mature manhood. Prolonged rest and travel in foreign lands failed to bring back the vigor and ambition of youth. Calmly estimating the factors in the problem of life, he realized the inevitable result of the morbific changes going on in his system, and bravely awaited the fulfilment of the inevitable law which consigns his body to mother earth and his soul to its eternal rest.

Finally, it is resolved that this Society attend the funeral of our deceased brother, and a copy of this memorial be transmitted to his family.

THOMAS LOTHROP,
F. W. BARTLETT,
H. R. HOPKINS.

Remarks were made in support of the memorial and eulogistic of the life and character of Dr. Hill by Drs. Thomas Lothrop, Lucien Howe, F. W. Bartlett, H. E. Hayd, H. R. Hopkins, and C. A. Ring.

Dr. J. B. ANDREWS submitted the following in writing:

I desire to make a few remarks upon the memorial which has been presented to the society regarding the death of Dr. John D. Hill. It has been my good fortune to know the doctor intimately for the past twelve years, during which time he has been a member of the Board of Managers of the Buffalo State Hospital; this acquaintance ripened into deep friendship, that gave me an insight into his life and character, which, perhaps, has fallen to the lot of but few here present. The knowledge of what he has done for the charities of the State and the city of Buffalo, was a revelation to me of the possession by him of the most admirable and lovable traits. He was a faithful friend and safe counselor, easily approached, always positive in his convictions and most earnest in their advocacy. Of his professional career, there are others here who have a better knowledge. His early professional life proved him to be a man who could depart from the routine of practice, and showed he had the courage to put in force his own views of treatment. To his patients he was something more than a physician,—he became to them a friend, and often stood in the place of a father. The families which he served became closely attached to him, and remained so until his last illness and death. His business ability was of a marked kind; he could intuitively see and appreciate any subject that was presented for his consideration. In overseeing the affairs of the institution, he had the same regard for economy, and the same zealous care of all its concerns that he had for his own personal interests. The patients at the hospital became much attached to him from his kind and gentle manners, and for the care which he exhibited for them; to the officers and employees he was always kind and courteous, and to them all his death is a personal loss. In his life he gave an example of the Christian physician, which cannot fail to make its impression upon all with whom he was associated.

The report of the committee was unanimously adopted by a rising vote.

The society then adjourned.

### OBSTETRIC CALENDAR.

Dr. Lingrand, *Le Concours Méd.*, gives the following table which will be found very convenient for making calculations, especially as it can be very readily learned:

| | | | | | |
|---|---|---|---|---|---|
| Jan | 3 | May | 6 | Sept. | 3 |
| Feb | 3 | June | 3 | Oct. | 3 |
| March | 5 | July | 4 | Nov. | 3 |
| April | 5 | Aug | 3 | Dec. | 4 |

To arrive at the 270 days, start at the day which marks the end of menstruation and count backwards three months, then subtract the number of days indicated in the table opposite the month in which the catamenia ceased. For example, the 24th of July being the day indicated above, we begin by counting backwards: June 24, May 24, April 24—April 24 less four days (the number opposite the month of July in the table)=April 20=270 days. To this may be added seven days, or better, from seven to seventeen days according as the fetal movements first perceived by the mother indicate a period more or less near the last day of menstruation.

Where February has twenty-nine days, as in the present year, and is included in the calculation, one day more should be subtracted.—*Medical Abstract.*

The physician who values his time and advice is the man who is appreciated.—*Ex.*

He who sells himself for nothing generally gets all he is worth.—*Ex.*

